# Ionic liquids with reversible photo-induced conductivity regulation in aqueous solution

**DOI:** 10.1038/s41598-023-40905-z

**Published:** 2023-08-23

**Authors:** Yige Zhang, Xiaowen Xie, Jianliang Liu, Boyuan Tang, Can Fang, Xiaoming Liu, Zhifeng Dai, Yubing Xiong

**Affiliations:** 1 Key Laboratory of Surface and Interface Science of Polymer Materials of Zhejiang Province, School of Chemistry and Chemical Engineering, Zhejiang Sci-Tech University, Hangzhou, 310018 People’s Republic of China; 2 Boya International Academy, Shaoxing, 312000 People’s Republic of China; 3 Zhejiang Institute of Standardization, Hangzhou, 310018 People’s Republic of China; 4 Longgang Institute of Zhejiang Sci-Tech University, Wenzhou, 325802 People’s Republic of China

**Keywords:** Electronic materials, Ionic liquids

## Abstract

Stimulus-responsive ionic liquids have gained significant attention for their applications in various areas. Herein, three kinds of azobenzimidazole ionic liquids with reversible photo-induced conductivity regulation were designed and synthesized. The change of electrical conductivity under UV/visible light irradiation in aqueous solution was studied, and the effect of chemical structure and concentration of ionic liquids containing azobenzene to the regulation of photoresponse conductivity were discussed. The results showed that exposing the ionic liquid aqueous solution to ultraviolet light significantly increased its conductivity. Ionic liquids with longer alkyl chains exhibited an even greater increase in conductivity, up to 75.5%. Then under the irradiation of visible light, the electrical conductivity of the solution returned to its initial value. Further exploration of the mechanism of the reversible photo-induced conductivity regulation of azobenzene ionic liquids aqueous solution indicated that this may attributed to the formation/dissociation of ionic liquids aggregates in aqueous solution induced by the isomerization of azobenzene under UV/visible light irradiation and resulted the reversible conductivity regulation. This work provides a way for the molecular designing and performance regulation of photo-responsive ionic liquid and were expected to be applied in devices with photoconductive switching and micro photocontrol properties.

## Introduction

Stimulus-responsive ionic liquids are these ionic liquids whose physical and chemical properties change correspondingly under external stimulation conditions, such as CO_2_^1–6^, temperature^7–10^, pH^11–14^, redox^15,16^, magnetism^17,18^, light^19–24^, etc. Due to their unique stimulus response capabilities, these ionic liquids can meet the needs of certain specific processes and hold significant promise for applications in controlled drug delivery, sensors, photovoltaic conversion and catalysis^25–29^. As an important stimulation method, light has the advantages of stable signals, precise stimulation sites, rapid switching, and the ability to be controlled remotely as well as the fact that no other substances are introduced into the stimulation process gives it unparalleled advantages in practical applications. As one of the common photoresponsive functional groups, azobenzene and its derivatives are one of the most widely used functional groups in photoresponsive ionic liquid research because of their high environmental sensitivity and reversibility, simple synthesis procedure, good photostability and reusability^30–35^. The addition of azobenzene groups to ionic liquids creates light-responsive ionic liquids with potential applications in various fields.

The regulation of solution conductivity is an important physicochemical parameter in electrolyte solutions. It has significant applications in optoelectronic modulation and self-healing electronic devices^36^. Zhang et al*.*^20^ synthesized a series of imidazole ionic liquids containing azobenzene group. The effect of UV illumination on the conductivity of these ionic liquids in organic solvents was investigated. The conductivity of the ionic liquid was minimally affected by UV light in acetone, chloroform, ether, and cyclohexane. However, it was reduced to varying degrees in dichloromethane, ethyl acetate, and tetrahydrofuran. It can be reduced to 0.9 times before the light, further using visible light irradiation, the conductivity of the system can return to the initial value. Since these azobenzene ionic liquids are hydrophobic, only their conductivity regulation in organic solvents was investigated, and its regulation efficiency in such systems was relatively low. A new class of inorganic azobenzene salts was also prepared by Wang et al.^37^ and the resulting azobenzene compounds exhibited significant and reversible light-responsive conductivity behaviour, which may be related to changes in polarity and ionisation processes caused by photoisomerisation, demonstrating the energy conversion from light to electricity with structural changes at the molecular level of inorganic azobenzene as molecular devices.

**Figure 1:**
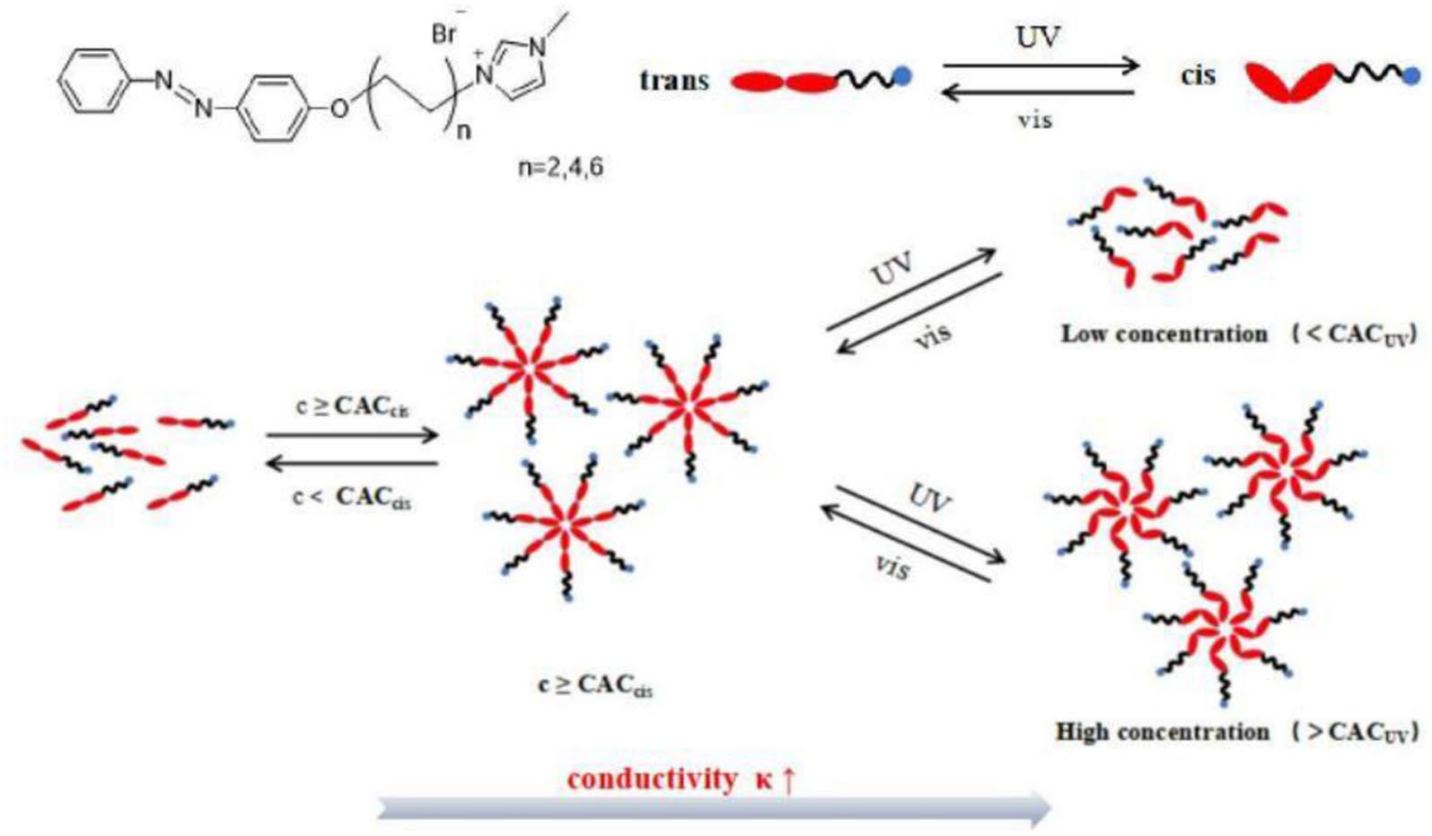
Illustration of the photo-regulation aggregation of the ILs in water.

In this study, three kinds of azobenzimidazole ionic liquids with different carbon chain length were designed and synthesized. The change of electrical conductivity under UV/visible light irradiation in aqueous solution was studied, and the chemical structure and concentration of ionic liquids containing azobenzene affecting the regulation of photoresponse conductivity were discussed. The results demonstrated that under the irradiation of ultraviolet light, the conductivity of ionic liquid aqueous solution was significantly increased. The photoinduced conductivity of ionic liquids with longer alkyl chains increased more significantly, up to 75.5%. Then under the irradiation of visible light, the electrical conductivity of the solution returned to the initial value. We hypothesized that the reversible conductivity regulation (as shown in Fig. 1) may be attributed to the formation/dissociation of ionic liquid aggregates in aqueous solution induced by the isomerization of azobenzene under UV/visible light irradiation.

## Experimental

### Materials

Aniline (99.5%) and 1,2-dibromoethane (99%) were purchased from Adamas-beta. Aniline needs to be further purified by vacuum distillation before using. Phenol (98%), 1,4-dibromobutane (97%), 1,6-dibromohexane (99%) and sodium nitrite (AR) were purchased from Meryer Chemical Technology (Shanghai). N-methylimidazole (99%) was purchased from Aladdin. Sodium hydroxide (AR), potassium carbonate (AR), sodium chloride (AR), methanol (AR), dichloromethane (AR), petroleum ether (AR) and ethyl acetate (AR) were purchased from National Pharmaceutical Group Chemical Reagent Co., Ltd. Hydrochloric acid (36.5%), diethyl ether (AR) and acetone (AR) were purchased from Hangzhou Shuanglin Chemical Reagent Co., Ltd. All other chemicals were purchased commercially and used as received unless otherwise specified.

### Synthesis

#### Synthesis of 4-hydroxyazobenzene (Azo-OH)

Aniline (5.0271 g, 54.00 mmol) and concentrated hydrochloric acid (37%) (16 mL) were dissolved in 25 mL water under ice bath and stirred for 30 min. NaNO_2_ (3.7100 g, 54.00 mmol) in 25 mL of water was slowly added at 0 °C and stirred for 1 h to get a diazonium salt solution. NaOH (5.0018 g, 125.00 mmol) and phenol (7.8015 g, 82.90 mmol) were dissolved in water (50 mL). Then, the diazonium salt solution was slowly dripped into it at 0 °C and stirred for 2 h. The precipitate was filtered and washed with H_2_O (200 mL) to get a dark yellow solid powder and dried under vacuum at 70 °C for 24 h. Yield: 9.88 g, 92.3%.

#### Synthesis of 2-bromoethyl-4-azophenyl ether (AzoC_2_Br)

4-hydroxyazobenzene (3.7234 g, 18.78 mmol), 1,2-dibromoethane (10.5320 g, 56.05 mmol) and potassium carbonate (6.9238 g, 50.00 mmol) were added to a round bottom flask containing 120 mL acetone. The reaction system refluxed at 70 °C under the protection of nitrogen atmosphere for 24 h, cooled to room temperature at the end of the reaction and dissolved potassium carbonate in the system with hydrochloric acid. The organic phase was extracted with dichloromethane (150 mL), then washed with distilled water and saturated salt water respectively. After the solvent was removed by rotary distillation, the solvent was further purified by column chromatography. Then the product was dried overnight at 60 °C to obtain orange powder solid. Yield: 2.76 g, 48.2%.

#### Synthesis of 1-methyl-3-[6-(4-phenylazophenoxy) ethyl] imidazole bromide ([AzoC_2_MIm]Br)

2-bromoethyl-4-azophenyl ether (3.0518 g, 10.00 mmol) and 1-methylimidazole (0.9852 g, 12.00 mmol) were added to a round bottom flask containing 30 mL MeOH. The reaction system refluxed at 70 °C under the protection of nitrogen atmosphere for 90 h, cooled to room temperature at the end of the reaction. The reaction system was precipitated in ether and dried overnight at 80 °C to obtain orange solid. Yield: 2.2811 g, 58.9%. The synthesis of 1-methyl-3-[6-(4-phenylazophenoxy) butyl] imidazolium bromide ([AzoC_4_MIm]Br) and 1-methyl-3-[6-(4-phenylazophenoxy) hexyl] imidazolium bromide ([AzoC_6_MIm]Br) was similar to that of [AzoC_2_MIm]Br.

### Characterization

Proton nuclear magnetic resonance spectroscopy (^1^H NMR) was recorded using a Brucker AM 400 MHz spectrometer at 25 °C. UV–Vis spectrum was recorded on a SHIMADZU, UV-2600 and the spectrum was recorded in a wavelength range from 250 to 500 nm. The aqueous solutions containing azobenzene ionic liquids with different concentrations were freeze-dried and the aggregate morphology of the freeze-dried samples was observed by SU8100 scanning electron microscope (SEM). Melting point temperatures were measured on a TA Instruments DSC 2000. The real-time resistance and capacitance signals were collected by a LCR meter (TH2830) under an AC voltage of 2.0 V and a sweeping frequency of 100 kHz. The electrical conductivity of ionic liquids at a specific concentration was measured using a DDS-307A conductivity meter at 25 °C. The temperature of the system was controlled within ± 0.01 °C using a DHC-05-8 low temperature constant temperature flume. Initially, an aqueous solution of ionic liquid containing azobenzene was exposed to ultraviolet light (365 nm, 30 mW cm^−2^) for 5 h at 25 °C, and its electrical conductivity was determined. Subsequently, it was exposed to visible light (indoor natural light) for another 5 h at the same temperature, and its electrical conductivity was measured again. The critical aggregation concentration (CAC) of an ionic liquid in water was determined using the conductometric titration method. A stock solution of the ionic liquid in water with a certain concentration was prepared. Deionized water of known volume was added to an empty beaker at 25 °C, followed by the addition of a known volume of the stock solution. After stabilization, the conductivity of the resulting aqueous solution containing azobenzene ions at different concentrations was measured.

## Results and discussions

**Figure 2:**
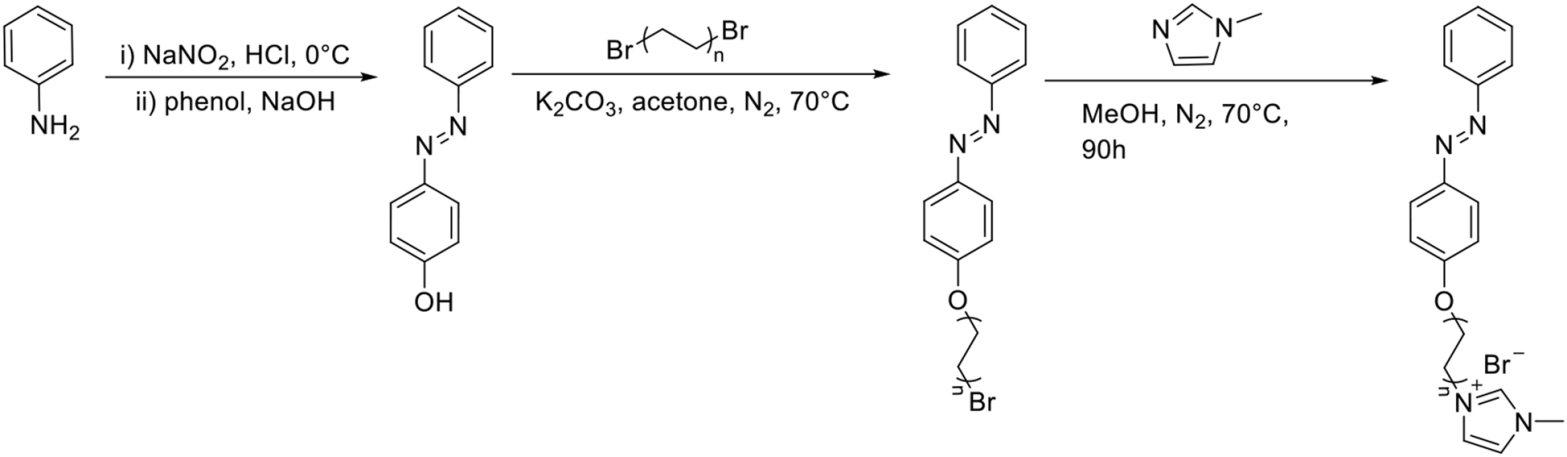
Synthesis of [AzoC_n_MIm]Br (n = 1, 2, 3) Ionic Liquids.

**Figure 3:**
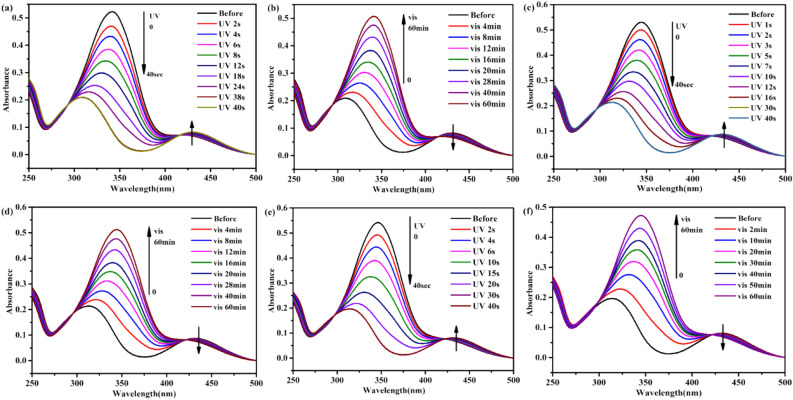
UV–vis spectra of azobenzene based ionic liquids: [AzoC_2_MIm]Br ((**a**) and (**b**)), [AzoC_4_MIm]Br ((**c**) and (**d**)) and [AzoC_6_MIm]Br (e and f) in aqueous solutions (3.3 × 10^−5^ mol L^−1^) under UV and visible light (indoor natural light) irradiations, respectively.

Figure 2 shows the synthesis process of the three kinds of ionic liquids. The obtained azobenzene ionic liquids were denoted as [AzoC_2_MIm]Br, [AzoC_4_MIm]Br and [AzoC_6_MIm]Br, respectively. The structure was confirmed by ^1^H NMR measurements (Figs. S1–S7). The melting point of ionic liquids is determined by DSC, which show that the melting point of [AzoC_n_MIM]Br was 121.10 °C, 133.16 °C and 142.44 °C, respectively (Fig. S8). The reversible photoisomerization of *trans* and *cis* azobenzene compounds under light irradiation was monitored by UV–Vis spectra (Fig. 3). Taking [AzoC_2_MIm]Br as an example (Fig. 3a), the obtained azobenzene ionic liquids were in a stable *trans* state and shown a strong π → π* band at 344 nm. The typical strong π → π* absorption band at 344 nm and wide n → π* absorption band at 430 nm were observed in UV–Vis spectra, which were corresponding to the *trans* isomerization and *cis* isomerization of azo functional groups, respectively. After UV irradiation, the π → π* bands of *trans* isomers decreased, while the n → π* bands of *cis* isomers at 430 nm increased. Upon visible light illumination (indoor natural light), the strength of adsorption band at 430 nm increase with the decrease of adsorption band at 344 nm, indicated the change of *cis* isomerization to *trans* (Fig. 3b). Therefore, the reversible photoisomerization of [AzoC_2_MIm]Br in aqueous solution was realized at room temperature. Similar isomerization behavior of [AzoC_4_MIm]Br and [AzoC_6_MIm]Br in aqueous solution were also observed as shown in the Fig. 3c–f.

**Figure 4:**
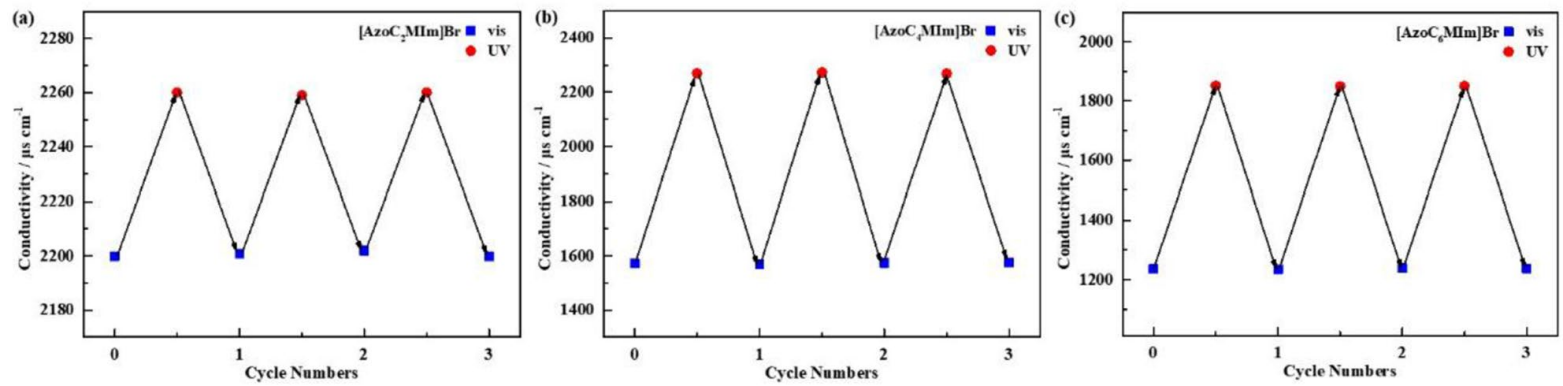
Reversible conductivity change of (**a**) [AzoC_2_MIm]Br, (**b**) [AzoC_4_MIm]Br, (**c**) [AzoC_6_MIm]Br aqueous solution (3.0 × 10^−2^ mol L^−1^) under alternating UV and visible light (indoor natural light) irradiation for 5 h at 25 °C.

Then the photo-modulated ionic conductivity of azobenzene ionic liquids in aqueous solution were studied. As shown in Fig. 4a, the ionic conductivity of [AzoC_2_MIm]Br-*trans* in aqueous solution (3.0 × 10^−2^ mol L^−1^) was 2200 μs cm^−1^, the [AzoC_2_MIm]Br-*trans* in aqueous solution isomerized to [AzoC_2_MIm]Br-*cis* after UV irradiation at 25 °C for 5 h, and the conductivity increased to 2260 μs cm^−1^. The increment percentage of conductivity △κ was 2.7% (△κ = (κ_t_ − κ_0_)/κ_0_; κ_0_, initial conductivity before UV irradiation, κ_t_, conductivity after 5 h UV irradiation). The electrical conductivity returned to the initial conductivity of 2201 μs cm^−1^ after 5 h visible light (indoor natural light) irradiation. These changing trends were almost keeping the same in the next two alternating irradiation. The initial conductivity of [AzoC_4_MIm]Br-*trans* and [AzoC_6_MIm]Br-*trans* in aqueous solution were 1571 μs cm^−1^ and 1237 μs cm^−1^, respectively. After the UV irradiation, the conductivity of [AzoC_4_MIm]Br and [AzoC_6_MIm]Br increased to 2270 μs cm^−1^ and 1852 μs cm^−1^ with △κ of 44.5% and 59.8%, respectively (Fig. 4b and c). The conductivity returned to its initial values after visible light exposure, indicating the reversible nature of this transformation.

The initial conductivity of the three ionic liquids containing azobenzene was [AzoC_2_MIm]Br > [AzoC_4_MIm]Br > [AzoC_6_MIm]Br at the same concentration (3.0 × 10^−2^ mol L^−1^). The solubility of ionic liquids in water may be influenced by this factor, with shorter carbon chain ionic liquids exhibiting higher solubility. Therefore, at the same concentration, the ionic liquid aqueous solution with short carbon chain had relative high electrical conductivity. And the △κ of [AzoC_4_MIm]Br and [AzoC_6_MIm]Br were much larger than that of [AzoC_2_MIm]Br.

Furthermore, the effect of ionic liquids concentration on the variation of photo-induced conductivity regulation were also explored, and the increment percentage of conductivity △κ of these materials at different concentrations were determined. As shown in Table 1, the electrical conductivity of the three aqueous solutions containing azobenzene ionic liquids changed after UV irradiation only when they reached a certain concentration. For [AzoC_2_MIm]Br relative higher concentration are required to achieve the light-induced conductivity regulation, while the [AzoC_6_MIm]Br can achieved this phenomenon at relative low concentration. Obviously, with the increase of the concentration of azobenzene ionic liquids in aqueous solution, the increment percentage of conductivity △κ of the three kinds ionic liquids before and after UV irradiation had a tendency to increase. In the range of this work, the biggest increment percentage of conductivity △κ before and after UV irradiation was [AzoC_6_MIm]Br aqueous solution at the concentration of 0.025 mmol L^−1^, the values of △κ was up to 75.5% which was much higher than those of reported literature results.

**Table 1:** The increment percentage of conductivity (△κ) of azobenzene ionic liquids in water increases with the concentration at 25 °C.

IL/mol L^−1^	△κ/%							
	0.003	0.005	0.015	0.025	0.030	0.050	0.070	0.080
[AzoC_2_MIm]Br	0	0	0	0	2.7	22.8	40.9	29.8
[AzoC_4_MIm]Br	0	0	11.4	35.9	44.5	63.6	49.2	42.6
[AzoC_6_MIm]Br	0	6.1	59.0	75.5	59.8	–	–	–

△κ = (κ_t_ − κ_0_)/κ_0_; κ_0_, electrical conductivity of solution without UV irradiation; κ_t_, electrical conductivity of the solution irradiated by ultraviolet light for 5 h.

–The concentration of saturated solution has been exceeded and no measured data have been obtained.

**Figure 5:**
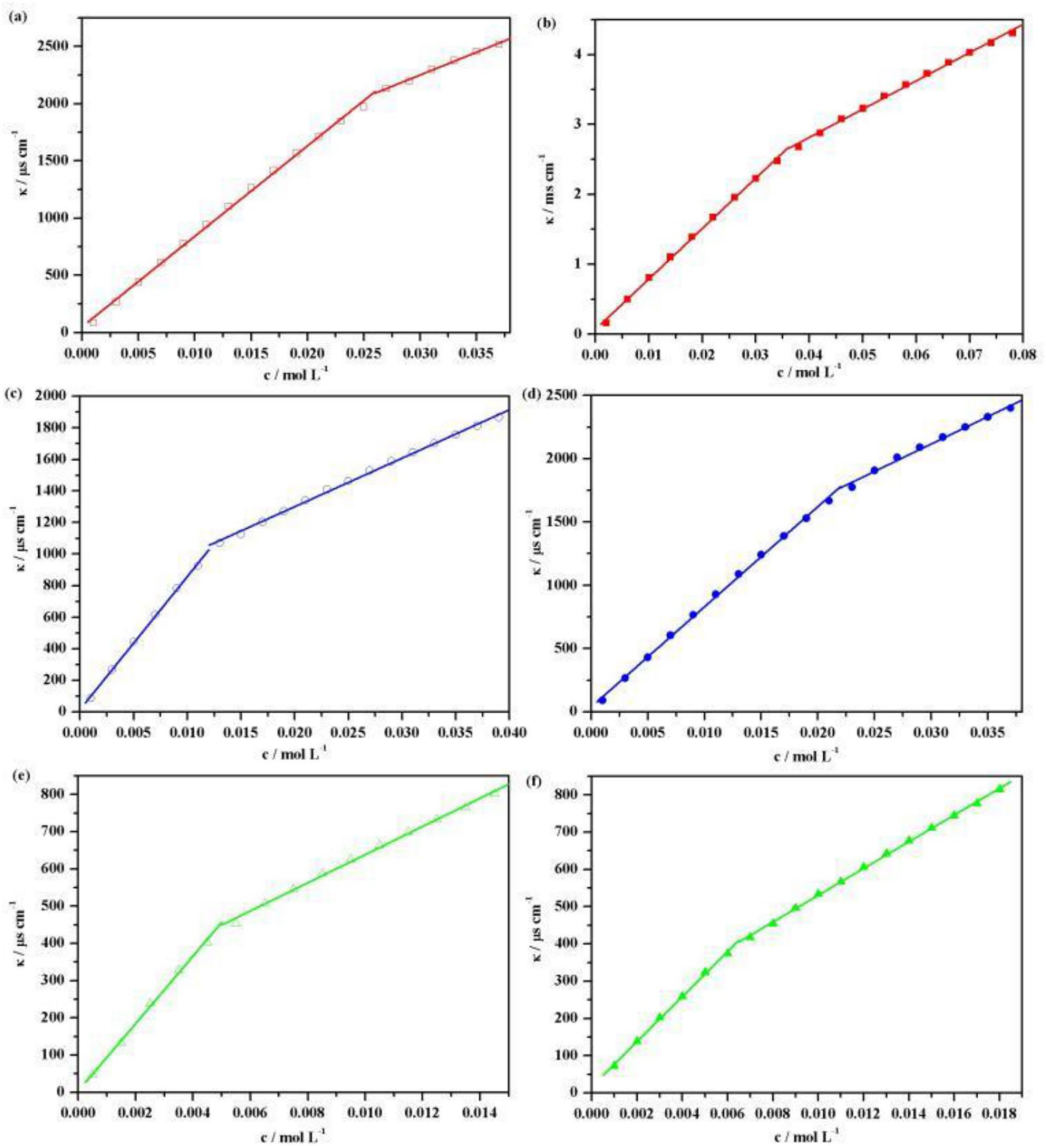
The conductivity of azobenzene ionic liquids in aqueous solutions as a function of the concentration at 25 °C. (**a**) [AzoC_2_MIm]Br before UV irradiation; (**b**) [AzoC_2_MIm]Br after UV irradiation for 5 h; (**c**) [AzoC_4_MIm]Br before UV irradiation; (**d**) [AzoC_4_MIm]Br after UV irradiation for 5 h; (**e**) [AzoC_6_MIm]Br before UV irradiation; (**f**) [AzoC_6_MIm]Br after UV irradiation for 5 h.

To better understand how the reversible photo-induced conductivity of azobenzene ionic liquids in aqueous solution is regulated, we measured their critical aggregation concentrations (CAC) using conductometric titration. The change of conductivity with concentration of these ionic liquids are shown in Fig. 5. The abscissa concentration of the intersection of the two linear segments represent the critical aggregation concentration (CAC) of the azobenzene ionic liquid in aqueous solution as presented in Table 2.

**Table 2:** The CAC value of ILs Aqueous solution at 25.0 °C before and after UV irradiation.

IL	CAC / mol L^−1^	
	Before UV	After UV
[AzoC_2_MIm]Br	0.0256	0.0356
[AzoC_4_MIm]Br	0.0125	0.0217
[AzoC_6_MIm]Br	0.0050	0.0063

Combined with analysis of the data in Tables 1 and 2, we found that only when the concentration of ionic liquids reached the CAC, the photo-induced electrical conductivity regulation happened before and after illumination. We believe that this may be due to the formation and dissociation of aggregates during the illumination process. When the concentration of ionic liquids in aqueous solution reached the CAC, they formed aggregates in water. The formation of aggregates was bound to reduce the electrical conductivity of the ionic liquid solution. Thus, after the UV irradiation, the hydrophobic *trans*-isomer of azobenzene was transformed into hydrophilic *cis*-isomer. Due to the hydrophilic property of *cis*-isomers, they show poor solve-ophobic action and was not conducive to the formation of aggregates and resulted the increasing of conductivity values.

**Figure 6:**
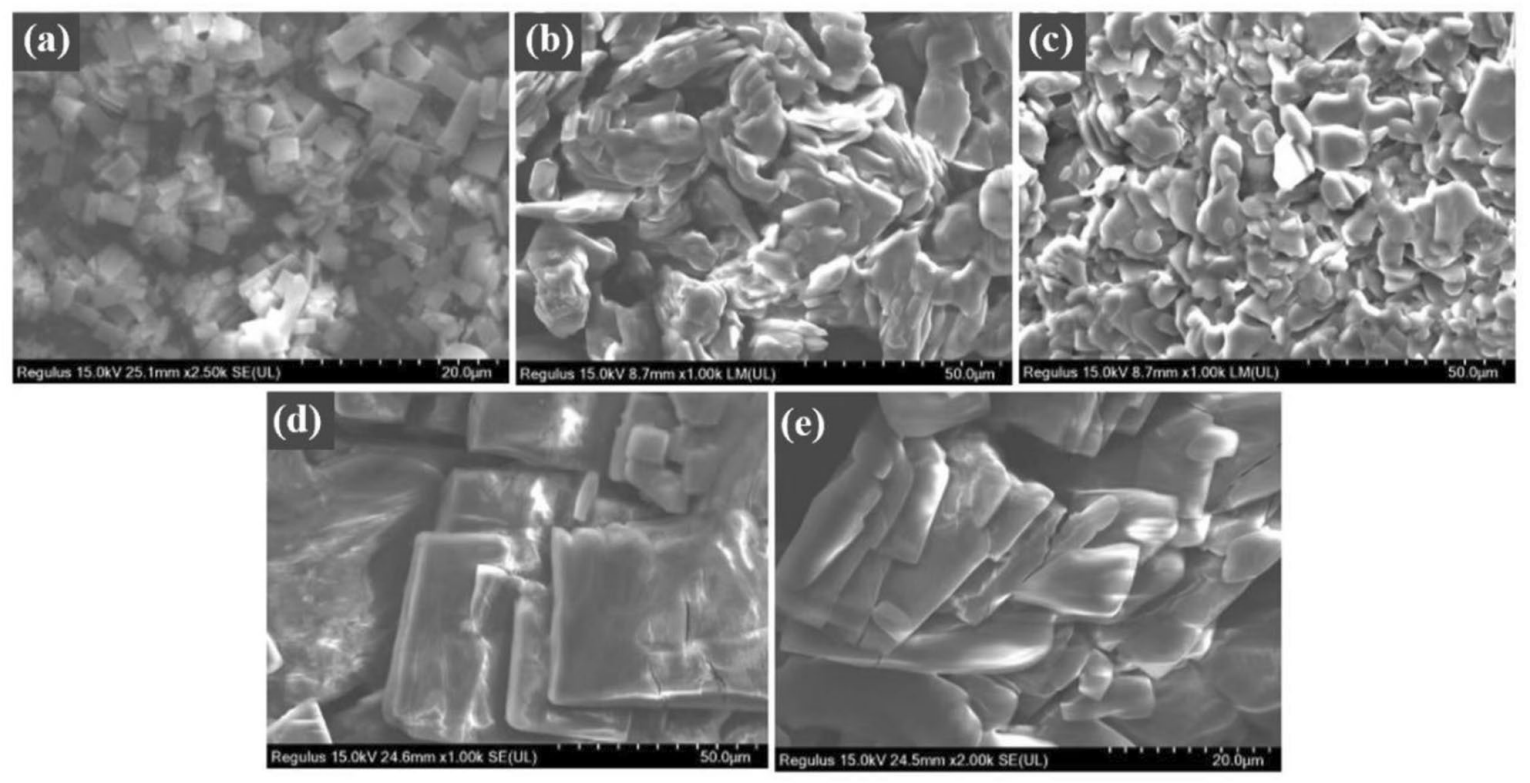
The SEM images of freeze-dried [AzoC_4_MIm]Br powders with different concentrations and configurations: (**a**) 0.005 mol L^−1^ before UV irradiation ([AzoC_4_MIm]Br-trans); (**b**) 0.018 mol L^−1^ before UV irradiation ([AzoC_4_MIm]Br-trans); (**c**) 0.018 mol L^−1^ after UV irradiation ([AzoC_4_MIm]Br-cis); (**d**) 0.080 mol L^−1^ before UV irradiation ([AzoC_4_MIm]Br-trans); and (**e**) 0.080 mol L^−1^ after UV irradiation ([AzoC_4_MIm]Br-cis).

This hypothesis was further confirmed by the SEM images of these ionic liquids with different concentrations before and after UV irradiation. Taking [AzoC_4_MIm]Br as an example, the CAC of *trans-*[AzoC_4_MIm]Br was 0.0125 mol L^−1^. Therefore, it can be inferred that at a concentration of 0.005 mol L^−1^, which is lower than the CAC, the ionic liquids should not aggregate in water before UV irradiation. As can be seen in Fig. 6a, the morphology of ionic liquid after freeze-dried showed dispersed small size particles. Figure 6b shows the observation of much larger worm-like particles with entangled sizes when the concentration increased to 0.018 mol L^−1^. After the aqueous solution was irradiated by ultraviolet light, the morphology of the ionic liquid changed to small particles indicated the dissociation of the aggregate due to the configuration change of *trans*-azobenzene to *cis-*isomer*,* as show in Fig. 6c. This was also the direct reason why ultraviolet light can increase the electrical conductivity of aqueous solution. When the concentration was further increased to 0.08 mol L^−1^, which was much higher than the CAC of *trans*-[AzoC_4_MIm]Br and also higher than the CAC of *cis*-[AzoC_4_MIm]Br (0.0217 mol L^−1^), it can be seen that a larger lamellar morphology of *trans*-azobenzene ionic liquid was observed as show in Fig. 6d. Even after UV irradiation, it still maintained an obvious aggregate morphology and corresponded to the decreasing of the increment percentage of conductivity △κ after UV irradiation (Fig. 6e). This may because at a concentration higher than the CAC of *cis*-azobenzene ionic liquid in aqueous solution, the ionic liquids still can form aggregates and resulted the decrease of △κ. Obviously, the photo-induced reversible conductivity regulation of azobenzene ionic liquids in aqueous solution is essentially the result of the formation and dissociation of ionic liquid aggregates caused by the photo-induced isomerization of azobenzene in water. When the concentration of ionic liquids reached the CAC of *trans*-azobenzene ionic liquid, the solution conductivity can be reversibly adjusted by light. If the concentration is higher than the CAC of *cis*-azobenzene ionic liquid, the increasing efficiency of solution conductivity would be weakened.

**Figure 7:**
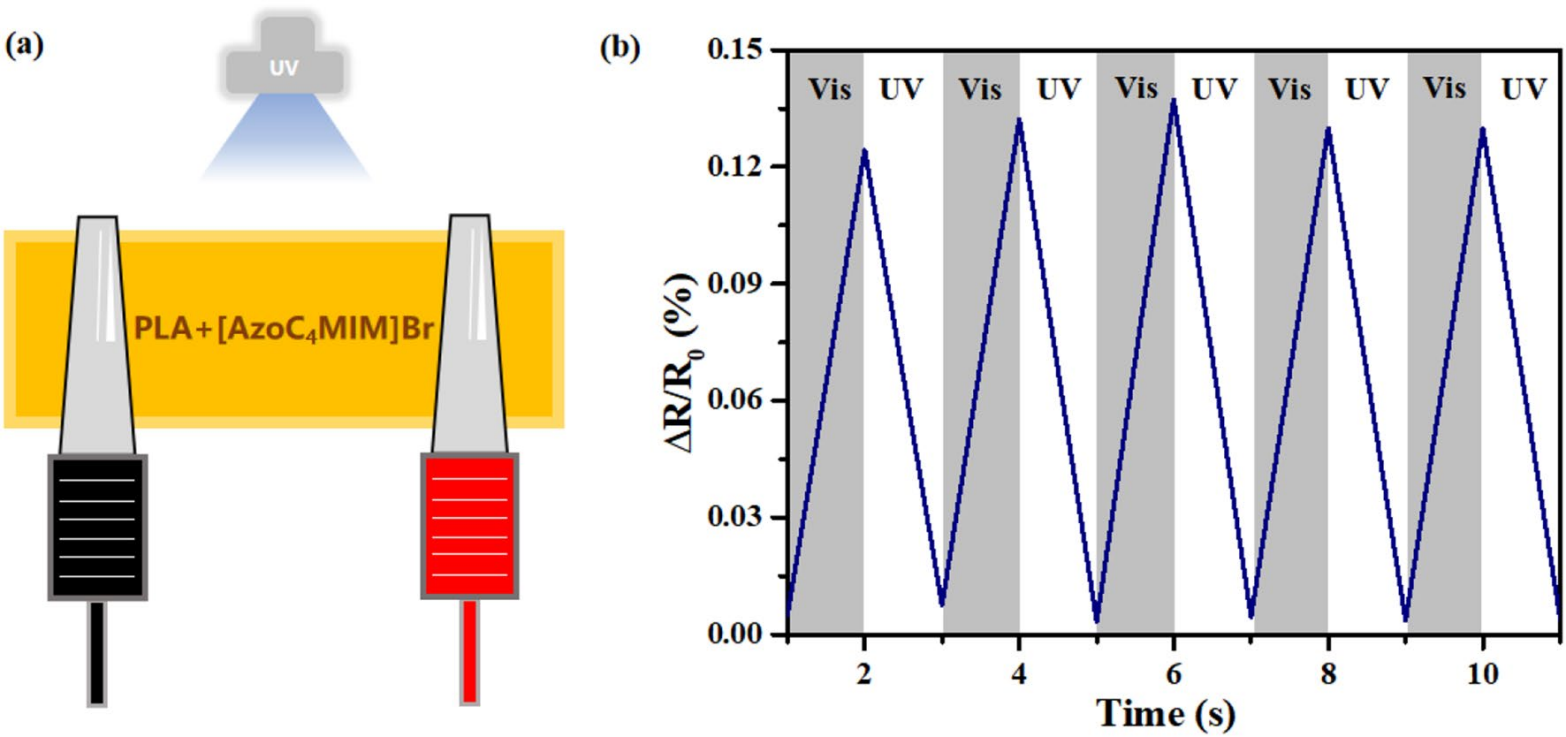
(**a**) Schematic illustration of the conductor and capacitor tests under UV/vis illumination. For the fabrication of capacitor, Ni and silicone were used as the electrodes and dielectric layer, respectively. (**b**) The change of resistance signal detected by PLA-IL sensor. (UV: λ = 365 nm, 30 mW cm^−2^; Vis: indoor natural light).

To further explored the potential application of these ionic liquids, we prepared a novel sensor material PLA-IL through the combination of PLA and [AzoC_4_MIM]Br (Fig. 7a). PLA is a biocompatible dynamic polymer that is often used as an adhesive to modify other conductive materials and is suitable for the research and development of wearable electronic products and sensors. The material was prepared by the addition of [AzoC_4_MIM]Br to PLA (nPLA:nIL = 10:1), and a small amount of ethanol was added as solvent. After the sample was completely dissolved, it was moved into the mold and finally heated in an oven at 80 °C for 6 h.

The light-induced conductivity regulation of PLA-IL was tested and presented in Fig. 7b. Firstly, the conductive behavior of PLA was characterized. We found that UV irradiation has no effect on the resistance of PLA. However, for the PLA-IL it was found that the resistance value decreased significantly after the UV light irradiation. The process was rapid, and the resistance value could be almost restored to the tarted value even after 5 times alternate irradiation. Our work ensures its potential application in resistance-responsive flexible sensors and provides great potential for new stimulus-responsive electronic devices used for the UV light detection.

## Conclusions

In summary, a series of azobenzimidazole ionic liquids with reversible photo-induced conductivity regulation in aqueous solution have been developed. By alternating the UV/visible light irradiation, the conductivity of ionic liquid aqueous solution was significantly changed and the conductivity of ionic liquids with longer alkyl chains could increase much significantly with conductivity values up to 75.5%. This was due to the dissociation of the *trans*-isomeric aggregates of ionic liquids to *cis*-isomers in aqueous solution by the UV irradiation, and improved their electrical conductivity. This work provides a way for the molecular designing and performance regulation of photo-responsive ionic liquid and were expected to be applied in devices with photoconductive switching and micro photocontrol properties.

### Supplementary Information

Supplementary Figures.

## Data Availability

The datasets used and/or analysed during the current study are available from the corresponding author on reasonable request.
