# Supplementary material for: Ionic liquids with reversible photo-induced conductivity regulation in aqueous solution
Source: Sci Rep. 2023 Aug 23;13:13766. doi: 10.1038/s41598-023-40905-z (PMC10447455; doi:10.1038/s41598-023-40905-z)
Supplement: Supplementary file 1 — Supplementary Figures. [file 41598_2023_40905_MOESM1_ESM.docx]

**Supporting Information**

**Ionic Liquids with Reversible Photo-induced Conductivity Regulation in Aqueous Solution**

Yige Zhang,^a+^ Xiaowen Xie,^a+^ JianLiang Liu,^a+^ Boyuan Tang,^b^ Can Fang,^a^ Xiaoming Liu,^c^ Zhifeng Dai,^a,d^* and Yubing Xiong,^a,d^*

*^a^ Key Laboratory of Surface & Interface Science of Polymer Materials of Zhejiang Province, Department of Chemistry, College of Science, Zhejiang Sci-Tech University, Hangzhou 310018, PR China.*

*^b^ Boya International Academy, Shaoxing 312000, PR China.*

*^c^ Zhejiang Institute of Standardization, Hangzhou 310018, PR China.*

*^d^ Longgang Institute of Zhejiang Sci-Tech University, Wenzhou 325802, PR China.*

*^+^ These authors contribute equal to this work.*

**Figure S1.** ^1^H NMR spectrum (400 MHz) of 4-hydroxyazobenzene (Azo-OH) in DMSO-*d*^6^.

**Figure S2.** ^1^H NMR spectrum (400 MHz) of 2-bromoethyl-4-azophenyl ether (AzoC_2_Br) in DMSO-*d*^6^.

**Figure S3.** ^1^H NMR spectrum (400 MHz) of 4-bromobutyl-4-azophenyl ether (AzoC_4_Br) in Chloroform-*d*.

**Figure S4.** ^1^H NMR spectrum (400 MHz) of 6-bromohexyl-4-azophenyl ether (AzoC_6_Br) in DMSO-*d*^6^.

**Figure S5.** ^1^H NMR spectrum (400 MHz) of 1-methyl-3-[2-(4-phenylazophenoxy) ethyl] imidazole bromide ([AzoC_2_MIm]Br) in DMSO-*d*^6^.

**Figure S6.** ^1^H NMR spectrum (400 MHz) of 1-methyl-3-[4-(4-phenylazophenoxy) butyl] imidazole bromide ([AzoC_4_MIm]Br) in DMSO-*d*^6^.

**Figure S7.** ^1^H NMR spectrum (400 MHz) of 1-methyl-3-[6-(4-phenylazophenoxy) hexyl] imidazole bromide ([AzoC_6_MIm]Br) in DMSO-*d*^6^.


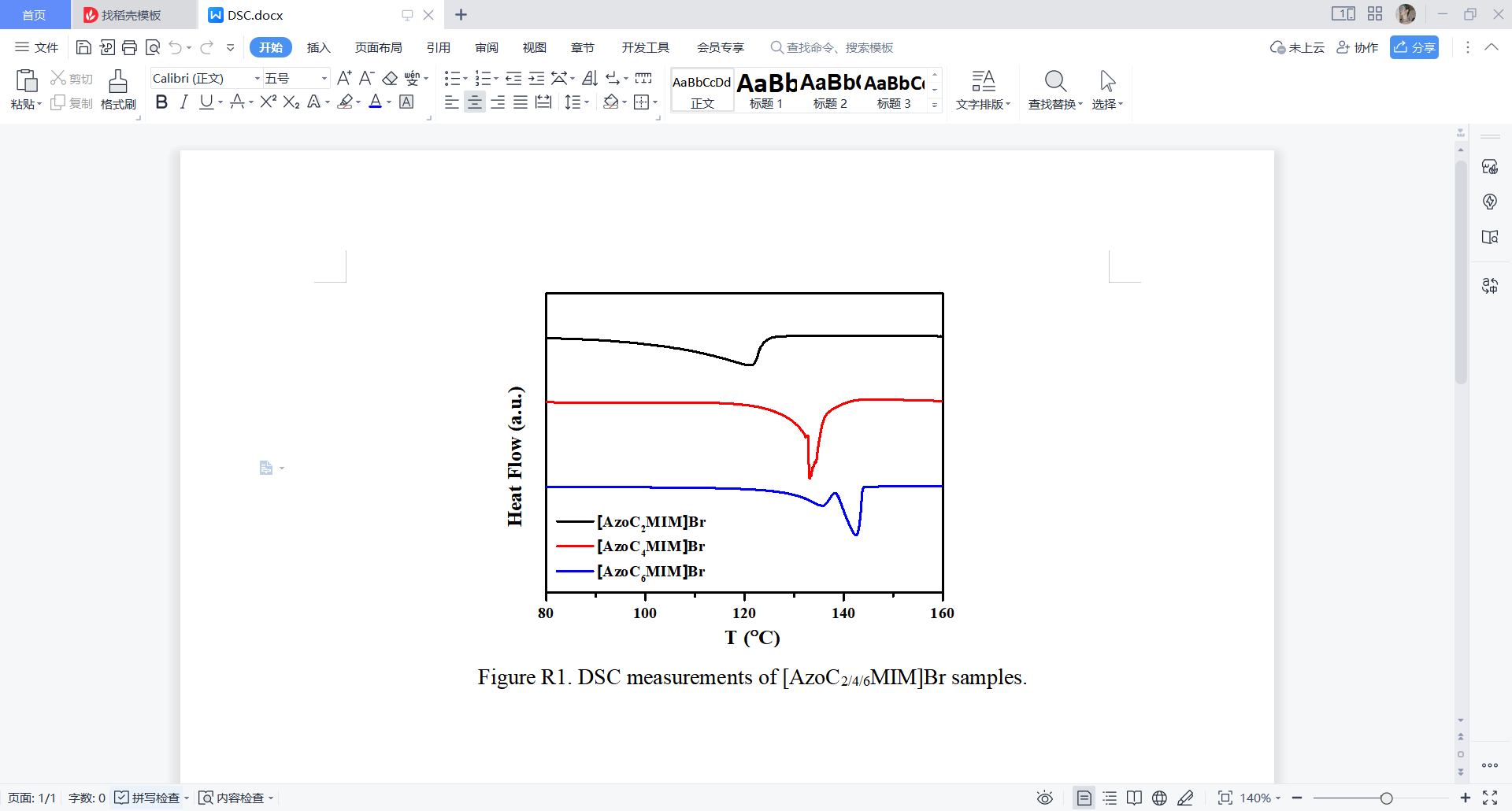


**Figure S8.** DSC measurements of [AzoC_2/4/6_MIM]Br samples.
